# The Effect of Ligament Modeling Technique on Knee Joint Kinematics: A Finite Element Study

**DOI:** 10.4236/am.2013.45A011

**Published:** 2014-05

**Authors:** Ata M. Kiapour, Vikas Kaul, Ali Kiapour, Carmen E. Quatman, Samuel C. Wordeman, Timothy E. Hewett, Constantine K. Demetropoulos, Vijay K. Goel

**Affiliations:** 1 Engineering Center for Orthopaedic Research Excellence (ECORE), University of Toledo, Toledo, USA; 2 Departments of Orthopaedics and Bioengineering, The University of Toledo, Toledo, USA; 3 Sports Health and Performance Institute (SHPI), The Ohio State University, Columbus, USA

**Keywords:** Finite Element, Knee, Biomechanics, Constitutive Model

## Abstract

Finite element (FE) analysis has become an increasingly popular technique in the study of human joint biomechanics, as it allows for detailed analysis of the joint/tissue behavior under complex, clinically relevant loading conditions. A wide variety of modeling techniques have been utilized to model knee joint ligaments. However, the effect of a selected constitutive model to simulate the ligaments on knee kinematics remains unclear. The purpose of the current study was to determine the effect of two most common techniques utilized to model knee ligaments on joint kinematics under functional loading conditions. We hypothesized that anatomic representations of the knee ligaments with anisotropic hyperelastic properties will result in more realistic kinematics. A previously developed, extensively validated anatomic FE model of the knee developed from a healthy, young female athlete was used. FE models with 3D anatomic and simplified uniaxial representations of main knee ligaments were used to simulate four functional loading conditions. Model predictions of tibiofemoral joint kinematics were compared to experimental measures. Results demonstrated the ability of the anatomic representation of the knee ligaments (3D geometry along with anisotropic hyperelastic material) in more physiologic prediction of the human knee motion with strong correlation (*r* ≥ 0.9 for all comparisons) and minimum deviation (0.9º ≤ *RMSE* ≤ 2.29°) from experimental findings. In contrast, non-physiologic uniaxial elastic representation of the ligaments resulted in lower correlations (*r* ≤ 0.6 for all comparisons) and substantially higher deviation (2.6° ≤ *RMSE* ≤ 4.2°) from experimental results. Findings of the current study support our hypothesis and highlight the critical role of soft tissue modeling technique on the resultant FE predicted joint kinematics.

## 1. Introduction

The knee is the largest and one of the most complex joints within the human body, consisting of both patellofemoral and tibiofemoral articulations. Anatomical structures such as ligaments, menisci and articular cartilage provide stability across the knee joint during functional daily activities. However, abnormalities due to age, injury, disease and other factors can affect biomechanical function of the knee joint. Mechanistic computational models, if properly validated, can serve as effective tools in parametric analyses, as well as population-based clinical studies. In particular, the use of finite element (FE) analysis has became progressively popular in the study of joint biomechanics as it allows for detailed analysis of the joint/tissue behavior under complex, clinically relevant loading conditions. FE methods have provided considerable insight into knee joint biomechanics, including ligament function, ligament reconstruction technique, and implant design. Due to inherent challenges associated with experiments (*in vivo* and *ex vivo*) and the associated high cost and time, FE analysis has long been recognized and trusted as a reliable alternative method in the study of human joints. Primary advantage of this numerical approach lies in precise control over boundary conditions, material properties and structural alterations in parametric studies. Moreover, the ligament forces/strains, contact forces/areas, and stress/strain distribution across soft and hard tissue structures are invaluable products of such a numerical approach, which are challenging, if not impossible, to obtain experimentally. The reliability of FE models strongly relies upon: a) appropriate representation of the geometry and assigned material properties, b) realistic simulation of interactions, constraints and boundary conditions, and finally c) thorough validation against experimental data.

Ligaments are soft connective tissues with a composite structure that connect bones together. As the main contributor to the overall joint stability, the mechanical function of these connective tissue structures is to guide normal joint motion and restrict abnormal joint movement. This is assisted by the topology of the articulating surfaces, muscle forces and other soft tissue constraints such as joint capsule. Physiologic characteristic of soft tissue material composition has always challenged the accuracy of the simplified numerical models of anatomical joints, specifically the knee joint which has been the scope of numerous studies due to its critical role in stability of human body during various physiological activities. A wide variety of modeling techniques have been utilized to model knee joint ligaments [1–12]. In majority of earlier FE studies of the knee joint, uniaxial discrete line elements (truss or spring) with simplified material properties were used to model ligaments [1–4,7]. Such an assumption of soft tissue geometry is associated with short- comings such as inability to predict non-uniform 3-dimensional (3D) stresses and strains across the tissue [6, 8,11]. Using Image processing techniques, ligaments were modeled with a 3D reconstructed geometry coupled with isotropic hyperelastic constitutive material models [9,10,12]. More recently, transversely isotropic hyperelastic constitutive models were developed and used to study knee ligaments [5,6,8,11].

Considering the critical role of ligaments in providing joint stability and associated assumptions with each modeling techniques in the characterization of tissue material properties, joint kinematics are expected to differ. However, the effect of selected constitutive model to simulate the ligaments on knee kinematics remains unclear. Hence, this study was designed to investigate alterations in knee joint kinematics under functional loading resulting from two different ligament modeling techniques: 1) uniaxial representation with isotropic non- linear elastic properties and 2) anatomic 3D representation with anisotropic hyperelastic properties. We hypothesized that anatomic representations of the knee ligaments with anisotropic hyperelastic material property will result in more realistic kinematics.

## 2. Methods

### 2.1. Model Development

**Figure.1:**
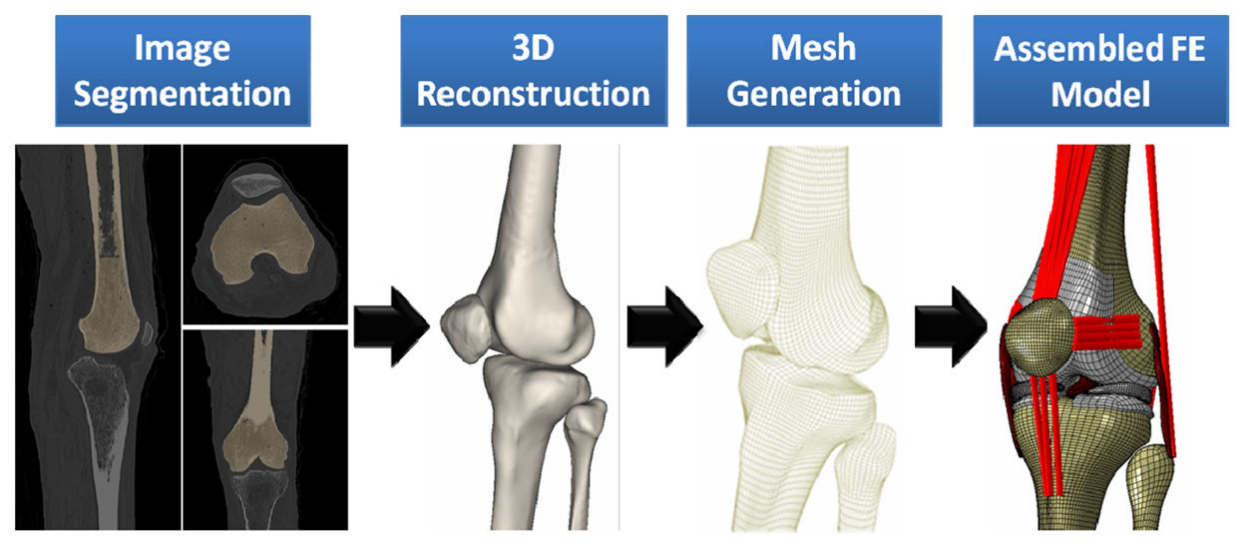
FE model development steps.

**Figure 2:**
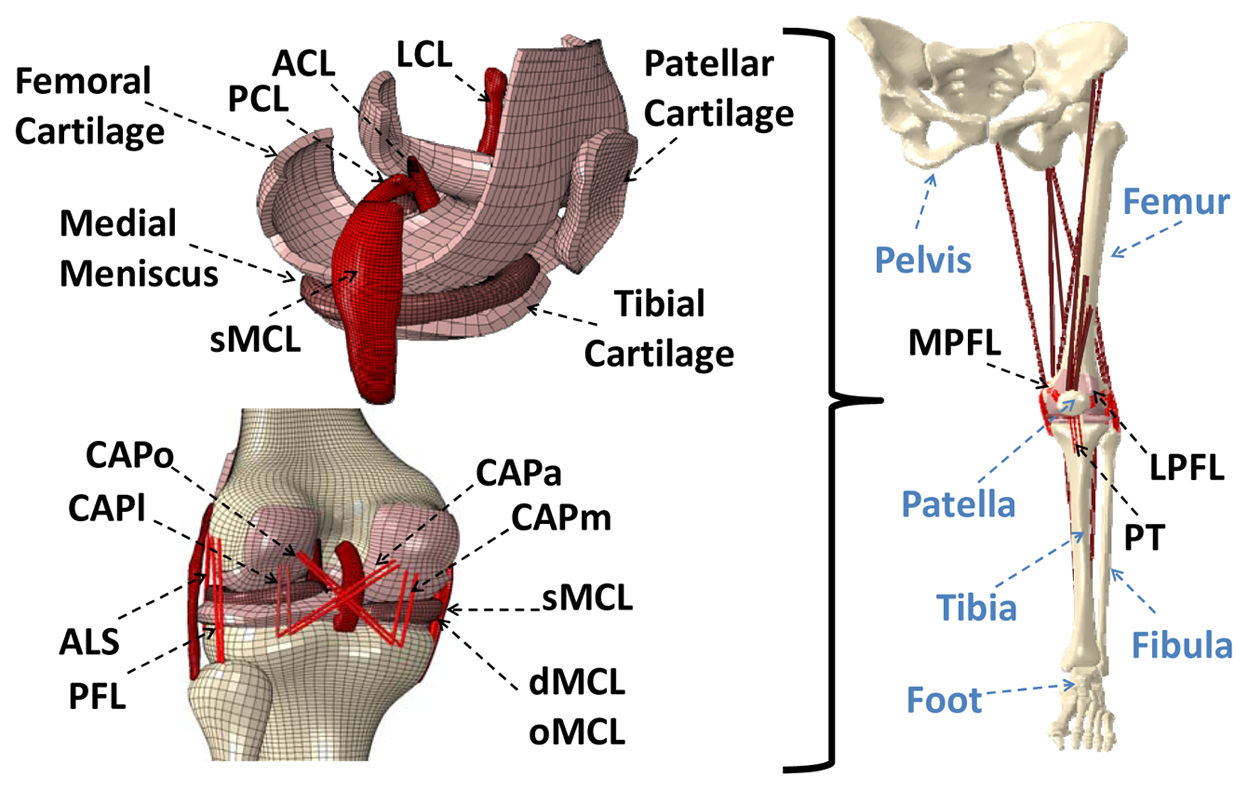
Developed FE model of lower extremity. (ACL: anterior cruciate ligament; PCL: posterior cruciate ligament; LCL: lateral collateral ligament; sMCL, dMCL and oMCL: superficial, deep and oblique bundles of medial collateral ligament; CAPm, CAPl, CAPo and CAPa: medial, lateral, oblique popliteal and arcuate popliteal bundles of posterior capsule; ALS: anterolateral structure; PFL: popliteofibular ligament; MPFL: medial patellofemoral ligament; LPFL: lateral patellofemoral ligament; PT: patellar tendon)

Following IRB approval, computerized tomography (CT) and magnetic resonance imaging (MRI) scans of a young adult female athlete’s lower limb (Age: 25 years, Height: 170 cm, Weight: 64.4 Kg) were used to capture bony and soft tissue geometry, respectively. Scans were obtained while the subject was supine with the leg in an unloaded neutral position. CT and MRI scans were co-registered for bony and soft tissue alignment. 3D geometry of the pelvis, leg (upper and lower) and foot segments were reconstructed from high resolution CT images in all three anatomical planes. Sagittal, coronal and axial MR images of the left knee were used to generate the 3D geometry of the knee articular cartilage, menisci, and knee cruciate and collateral ligaments. These geometries were then converted into solid 8-node hexahedral elements and subsequently imported into the ABAQUS FE package v6.11 (SIMULIA, Providence, RI, USA) to generate the FE model (Figure 1). While cruciate and collateral ligaments, articular cartilage and menisci were modeled as 3D structures, the rest of the simulated knee ligaments, joint capsule and muscle tendons were modeled as uniaxial truss elements (Figure 2).

To optimize computational expense, pelvis, proximal femur (from 10 cm above the joint line), distal tibia (from 10 cm below the joint line), fibula and foot were modeled as rigid bodies, while the remaining structures were considered deformable. Following assembly, proper material properties taken from literature were assigned to each segment [1,2,5,13–20]. Bones were modeled as linear elastic [21–25] with different moduli assigned to cortical and trabecular regions consistent with earlier FE studies of the human knee joint [2,13]. Tibiofemoral and patellofemoral articular cartilage were modeled as isotropic linear elastic [16]. Menisci were modeled as transversely isotropic linear elastic with different mechanical properties in circumferential, axial and radial directions [14,15,26]. Horn-meniscus attachment was simulated with multiple linear elastic truss elements [13].

Knee cruciate and collateral ligaments were modeled as incompressible anisotropic hyperelastic structures using the Holzapfel-Gasser-Ogden (HGO) material model [27]. HGO model is a hyperelastic, anisotropic material model that was developed to model the criss-crossed fibrous soft tissues like the illiac adventitia [16]. Briefly, isotropic non-collagenous ground matrix is modeled by the incompressible hyperelastic neo-Hookean component of the strain energy density (SED) function, whereas the transversely isotropic fibrous component is modeled by the following function developed by Gasser *et al*. [27]:

$$\bar{\Psi }\left(\bar{C},H_{i}\right)={\bar{\Psi }}_{g}\;\left(\bar{C}\right)+\sum_{i=1}^{2}\Psi_{fi}\;\left(\bar{C},H_{i}\;\left(a_{0i},\kappa \right)\right)$$

where **Ψ̄***_g_* and **Ψ̄***_fi_* are the respective isotropic and anisotropic components of the SED, ***a***_0_ is the mean orientation of the fibers, ***H* (*a***_0_,*κ*) is the structure tensor, and *κ* is the dispersion parameter for the fiber family. A statistical distribution function allows for a spatial distribution of the fiber orientation. Fibrous component of the SED supports tensile loads only and is defined as:

$${\bar{\Psi }}_{f}\;\left(\bar{C},H\right)=\frac{k_{1}}{2k_{2}}\left[\exp\left\{k_{2}{\left[\kappa {\bar{I}}_{1}+\left(1-3\kappa \right){\bar{I}}_{4}-1\right]}^{2}\right\}-1\right]$$

where *Ī*_1_= tr *C̄* is the first invariant of ***C̄*** and ***H*** is a generalized structure tensor defined as:

$$H=\kappa I+\left(1-3\kappa \right)\left(a_{0}⊗a_{0}\right)$$

The non-collagenous ground substance is modeled using the following incompressible isotropic neo-Hookean model:

$${\bar{\Psi }}_{g}\;(\bar{C})=\frac{1}{2}c\left({\bar{I}}_{1}-3\right)$$

Cruciate ligaments were modeled using two fiber families each in order to simulate bundles within ACL and posterior cruciate ligament (PCL) [17,18]. Both MCL (superficial bundle) and LCL were modeled using one family of fibers. Given the microstructure of the MCL and LCL, the HGO model was modified to account for a single family of fibers:

$$\bar{\Psi }\left(\bar{C},H\right)={\bar{\Psi }}_{g}(\bar{C})+{\bar{\Psi }}_{f}\;\left(\bar{C},H(a_{0},\kappa )\right)$$

**Figure 3:**
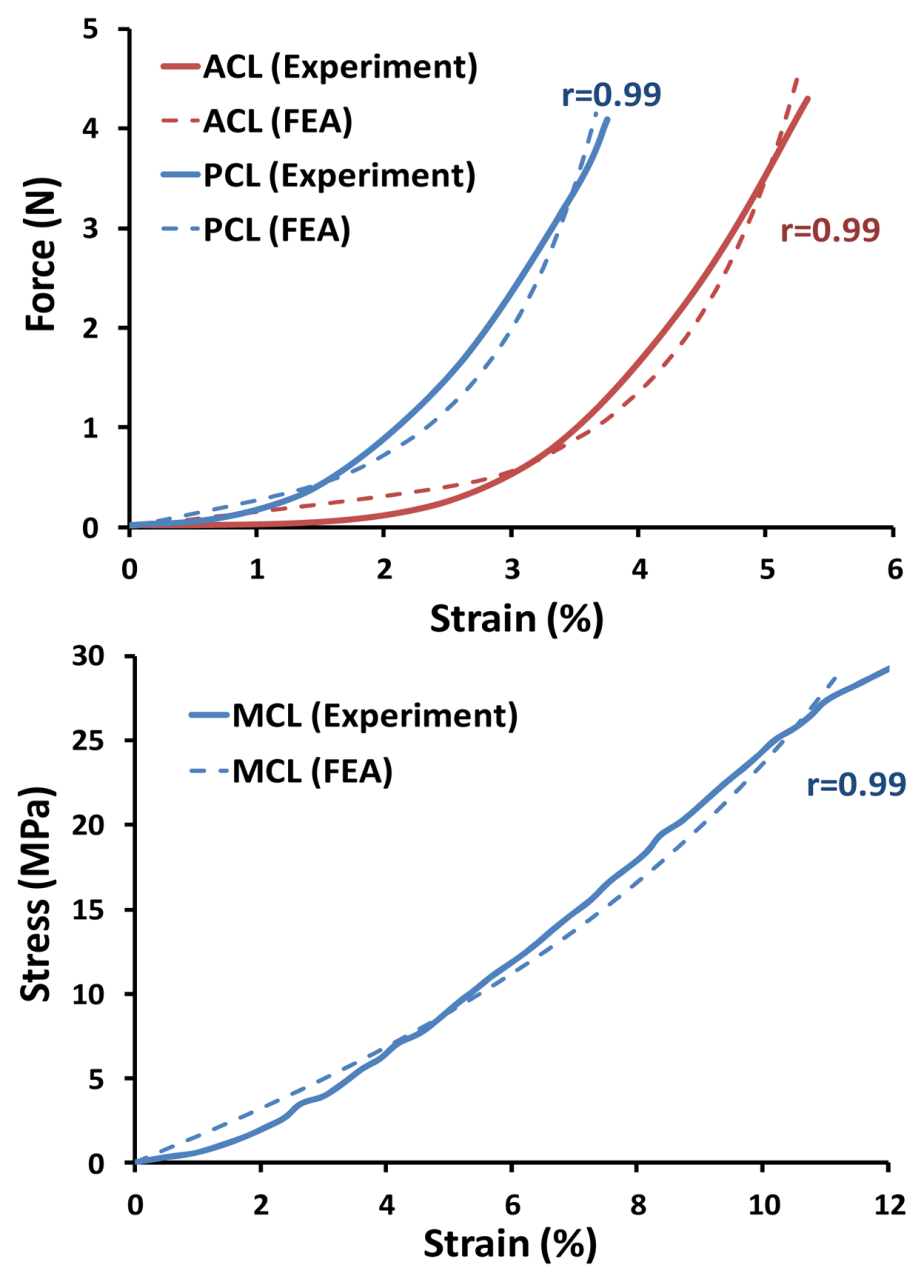
FE predictions vs. experimental data of the uniaxial tensile test for ACL, PCL (Top) and MCL (Bottom).

FE simulation of experimental uniaxial tensile tests along the longitudinal direction as per Butler *et al*. [28] for the ACL and PCL, and Quapp and Weiss [29] for the MCL were used to derive a series of coefficients for the constitutive model using a curve fitting technique (Figure 3). Coefficients for the lateral collateral ligament (LCL) were assumed to be identical to those of the MCL [5,11]. All other simulated knee ligaments were modeled as non-linear elastic, tension-only materials using truss elements with theoretically defined cross-sectional area. Further, 13 uniaxial truss-connector elements were used to simulate trans-knee muscle forces (Figure 2).

All other simulated knee ligaments were modeled as non-linear elastic, tension-only materials using truss elements with theoretically defined cross-sectional area. Further, 13 uniaxial truss-connector elements were used to simulate trans-knee muscle forces (Figure 2).

A frictionless surface-to-surface tangential contact with non-linear finite sliding interaction was used to simulate articular surfaces [3,11,13]. Since the current FE model was developed to investigate phenomena associated with knee biomechanics and relevant injuries, key knee joint soft tissue structures have been incorporated into the model. Both tibiofemoral and patellofemoral joints were simulated as six degree-of-freedom (DOF) joints with their motion defined by their surrounding soft tissue constraints and the topology of the articular surfaces. The hip and ankle joints were simplified as virtual ball-and-socket joints controlled by imported kinematic data, while optimizing for computational efficiency. The kinematics of the hip, knee and ankle joints were defined using the local coordinate systems proposed by Grood and Suntay [30]. Subsequently, the model was extensively validated against direct experimental measures of tibiofemoral kinematics, ACL and MCL strains and ti-biofemoral articular cartilage pressure distribution under a wide range of quasi-static and dynamic loading conditions [31].

### 2.2. Loading Profile

Four quasi-static loading conditions were simulated in order to compare the predicted FE kinematics with experimental measurements from an *ex vivo* study of 19 fresh frozen cadaveric legs [32,33]:

1) 0 to 50 Nm of knee abduction (at 25° of flexion), 2) 0 to 50 Nm of knee abduction + 20 Nm of internal tibial rotation (at 25° of flexion), 3) baseline (no external load, 0° – 90° of flexion), 4) 15 Nm of internal tibial rotation (0° – 90° of flexion), all under simulated muscle loads (*quadriceps*: 400 N *and hamstrings*: 200 N).

In order to study the effects of soft tissue material models, 3D reconstructed cruciate and collateral ligaments were substituted with multiple uniaxial representations (truss elements) with isotropic non-linear elastic material properties [2], while maintaining the same origins, insertions and initial orientation as the 3D model. Finally, the quasi-static simulations were repeated using uniaxial ligaments.

## 3. Results

### 3.1. Frontal Plane Kinematics (*Valgus Rotation*)

**Figure 4:**
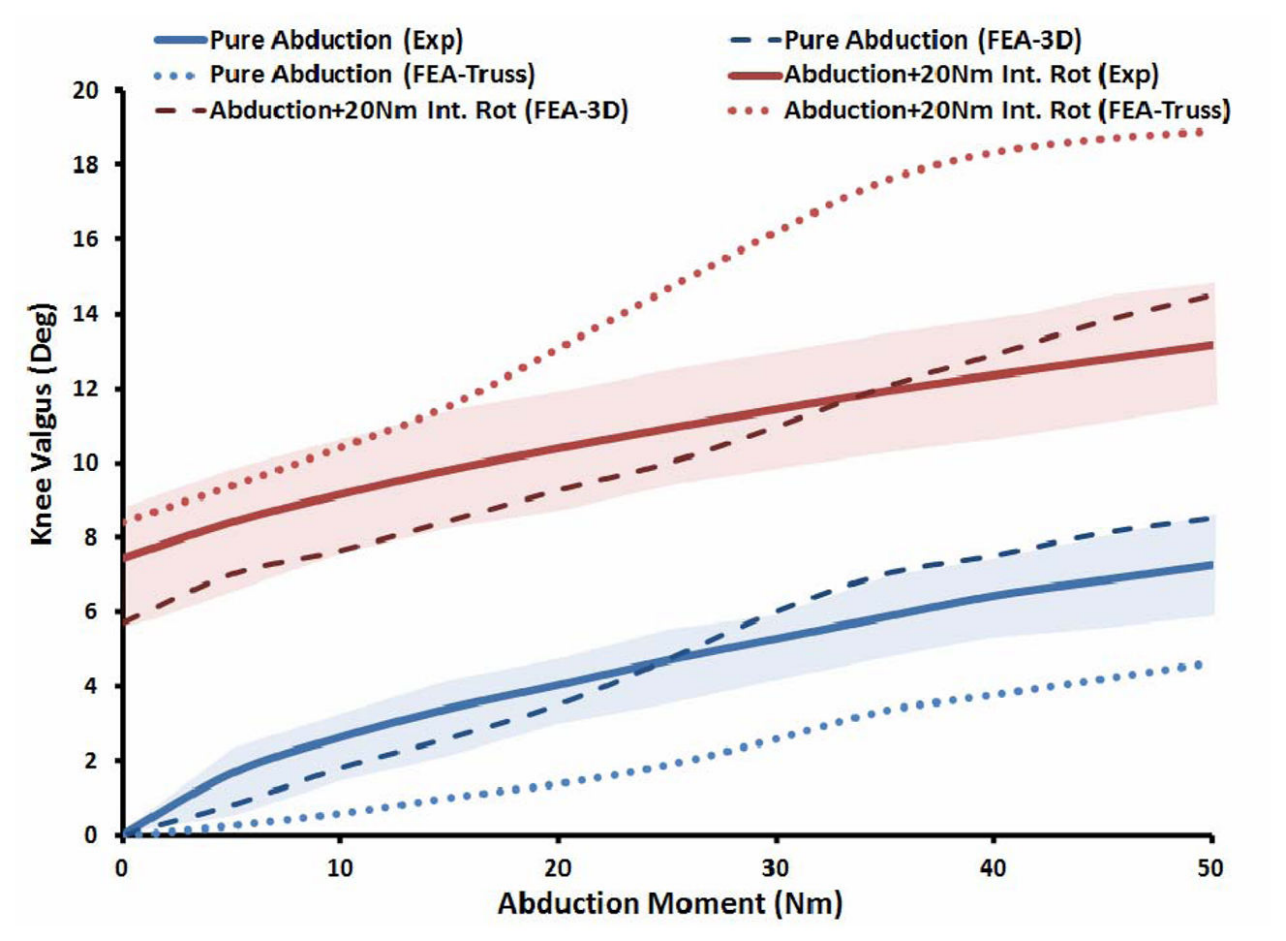
FE predictions Vs. experimental data for tibiofemoral frontal plane kinematics (Shaded area represent experimental 95% confidence intervals).

Both FE models resulted in similar frontal plane quality of motion as the experimental measurements under both single- and multi-axial loading conditions (Figure 4). Models also replicated coupled motion as observed in cadaveric experiments shown by knee valgus rotation under an additional internal tibial rotation moment of 20 Nm (Figure 4). The anatomic 3D representation of ligaments resulted in strong correlations (Pure abduction: *r =* 0.97, Combined abduction and internal rotation: *r =* 0.91) with minimum deviation (Pure abduction: *RMSE =* 0.9°, Combined abduction and internal rotation: *RMSE =* 1.2°) between FE model predictions and experimental measures of tibiofemoral frontal plane kinematics. Moreover, model predictions were within the range of 95% confidence intervals of average experimental measurements. In contrast, the uniaxial assumption coupled with simplified constitutive model of the knee ligaments resulted in substantially lower correlations (Pure abduction: *r =* 0.6, Combined abduction and internal rotation: *r =* 0.52 ) and higher deviation (Pure abduction: *RMSE =* 2.6°, Combined abduction and internal rotation: *RMSE =* 4.2 ) from the average experimentally quantified tibiofemoral kinematics. In addition to lower correlation and higher deviation from average experimental data, model predictions of joint kinematics were demonstrated to be outside the range of 95% confidence intervals of average experimental measurements (Figure 4).

### 3.2. Axial Plane Kinematics (*Internal Rotation*)

**Figure 5:**
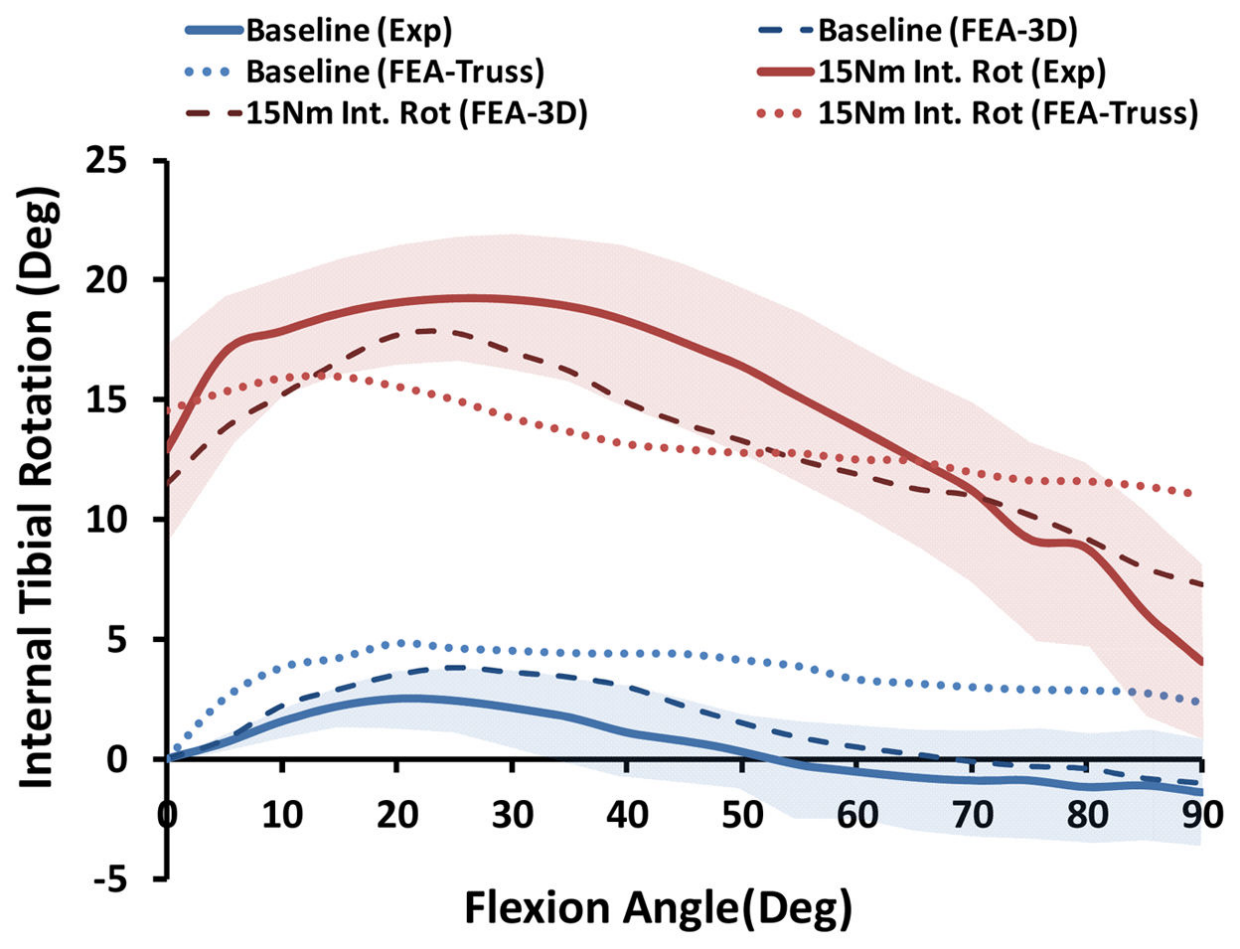
FE predictions Vs. experimental data for tibiofemoral axial plane kinematics (Shaded area represent experimental 95% confidence intervals).

Both FE models demonstrated similar trends as the experimental measurements under both single-and multi-axial loading conditions (Figure 5). Models also replicated knee joint screw-home mechanism [34] as observed in cadaveric experiments shown by internal tibial rotation during the early phase of flexion (Figure 5). The anatomic 3D representation of ligaments resulted in strong correlations (Baseline: *r =* 0.87, Internal rotation: *r =* 0.91) with minimum deviation (Baseline: *RMSE* = 1.1°, Internal rotation: *RMSE* = 2.2°) between FE model predictions and experimental measures of tibiofemoral axial plane kinematics. Moreover, model predictions were within the range of 95% confidence intervals of average experimental measurements. In contrast, the uniaxial assumption coupled with simplified constitutive model of the knee ligaments resulted in substantially lower correlations (Baseline: *r =* 0.58, Internal rotation: *r =* 0.47) and higher deviation (Baseline: *RMSE* = 3.2°, Internal rotation: *RMSE =* 3.7°) from the average experimentally quantified tibiofemoral kinematics. In addition to lower correlation and higher deviation from average experimental data, model predictions of joint kinematics were demonstrated to be outside the range of 95% confidence intervals of average experimental data (Figure 5).

## 4. Discussion

FE analysis is a powerful numerical technique that makes it feasible to investigate the biomechanical behavior of complex biological structures. During the past three decades, a large number of knee FE models with varying degrees of complexity, accuracy and functionality have been reported in the literature [1–12]. Simplified uniaxial representations of ligaments coupled with non-physiologic constitutive material models have been associated with the majority of these models [1–4,7]. More recent studies have used a 3D representation of knee ligaments with various degrees of anatomical and constitutive model complexity [5,6,8,9,11,12,28]. Song and colleagues developed a 3D FE model of the tibiofemoral joint which included 3D representation of the femur, tibia and ACL (with distinct AM and PL bundles) modeled as an isotropic hyperelastic material [9]. A similar model was developed by Gardiner and Weiss in order to study MCL biomechanics under functional loading [6]. They utilized a novel transversely isotropic, incompressible hyperelastic material model in order to simulate the MCL (superficial bundle) as a composite soft tissue structure [6]. Limbert *et al*. used a similar constitutive material model to study ACL biomechanics under passive tibial translation and flexion in a 3D FE model of an isolated ACL [8]. Others [5,11] have used similar constitutive modeling approaches with 3D simulations of key knee ligaments incorporated in 3D FE models of the knee joint. Despite substantial research efforts to develop soft tissue constitutive material models, little is known about the effects of such techniques on resultant joint function.

The purpose of the current study was to determine the effect of two most common techniques utilized to model knee ligaments on joint kinematics under functional loading conditions. A previously developed, extensively validated anatomic FE model of the knee developed from a healthy, young female athlete was used. FE models with 3D anatomic and simplified uniaxial representations of main knee ligaments (ACL, PCL, MCL and LCL) were recruited to simulate four quasi-static loading conditions as conducted in the cadaveric experiments.

The 3D anisotropic hyperelastic model resulted in a more physiologic prediction of the human knee motion under a range of single-and multi-planar functional loading conditions with strong correlation and minimal deviation from experimental data. In contrast, lower correlations in addition to notable deviations were observed using simplified uniaxial modeling technique. The current findings support our hypothesis and highlight the critical role of soft tissue modeling technique on resultant FE predicted joint kinematics. Anatomically accurate 3D representation of such structures coupled with structurally motivated constitutive models [27] facilitate implementation of realistic ligament mechanical properties such as finite deformation, anisotropy and non-linear incompressible fiber-reinforced structures. This approach also permits incorporation of realistic interactions between adjacent structures such as ligament-bone interaction that may also result in a more realistic simulation of lines of action as they vary with changes in joint orientation [8,11]. Moreover, anatomic representation of the ligament will also make it feasible to quantify local stress-strain distribution across the tissue, which is critical in study of ligament injury mechanisms.
